# Gender and socio-economic differences in physical activity and fitness among rural Bangladeshi adolescents: a cross-sectional study from the 15-year follow-up of MINIMat cohort

**DOI:** 10.1038/s41598-026-70291-1

**Published:** 2026-09-08

**Authors:** Mohammad Redwanul Islam, Christine Delisle Nyström, Paula Zart Krebs, Marie Löf, Syed Moshfiqur Rahman, Eva-Charlotte Ekström, Anisur Rahman

**Affiliations:** 1 Global Health and Migration Unit, Department of Women’s and Children’s Health, Uppsala University, Uppsala, 751 85 Sweden; 2 Institute of Environmental Medicine, Karolinska Institutet, Stockholm, Sweden; 3 Department of Medicine Huddinge, Karolinska Institutet, Stockholm, Sweden; 4 International Centre for Diarrhoeal Disease Research, Dhaka, Bangladesh

**Keywords:** Physical activity, Physical fitness, Accelerometry, Sedentary time, Grip strength, Gender disparity, LMIC, Rural adolescents, Health care, Medical research, Risk factors

## Abstract

Given the paucity of objective physical activity (PA) and fitness assessment among adolescents in low-income settings, the underlying gender and socio-economic disparities remain poorly understood. Hence, we aimed to examine the associations of gender, household wealth and maternal education with PA and fitness among adolescents of a rural birth cohort in southeast Bangladesh. This cross-sectional study utilized data collected during the 15-year follow-up of MINIMat (Maternal and Infant Nutrition Interventions in Matlab) cohort. In total, _2_465 adolescents completed the household survey, and 2253 attended the subsequent PA and fitness assessment. Wrist-worn, triaxial accelerometers (dynamic range ±8G) were used to estimate sedentary time (ST), and total and intensity-specific PA. Valid accelerometry data (≥ 5 days with ≥ 600 min/day awake wear time) were available for 1872 adolescents. Fitness indicators assessed were: handgrip strength, standing long jump distance, and aerobic capacity (VO_2max_). We fitted multiple multivariable-adjusted linear regression models and derived predicted means. About 58% adolescents spent ≥ 60 min/day in moderate-to-vigorous PA. After adjusting for socio-demographic variables, ST was higher among girls than boys (644 vs. 635 min/day; p < 0.001), in adolescents from the wealthiest than the poorest households (648 vs. 631 min/day; p < 0.001), and in adolescents with educated mothers compared to those having mothers without formal education (646 vs. 636 min/day; p = 0.005). While boys engaged in more vigorous PA (4 vs. 1 min(s)/day; p < 0.001), moderate PA was higher among girls (66 vs. 64 min/day; p = 0.007). Moderate-to-vigorous PA did not vary by gender, but was higher among socio-economically disadvantaged adolescents than their peers from wealthier households. Predicted levels of weight-normalized grip strength (0.66 vs. 0.51), standing long jump distance (160.2 vs. 126.3 cm), and VO_2max_ (34.8 vs. 33.2 mL/kg/minute) were higher among boys than girls. Adolescents from the wealthiest households had lower muscular fitness than those from the poorest households. We observed gender and socio-economic differences in PA and fitness, with girls and adolescents from relatively affluent and educated households demonstrating lower activity and fitness levels. Context-specific strategies tailored to gender and socio-economic disparities are needed to promote PA and fitness among rural adolescents in Bangladesh.

## Introduction

Insufficient physical activity (PA) accounted for an estimated 0.83 million deaths and 15.74 million disability-adjusted life years in 2019^1^. The latest estimates of population attributable fraction link insufficient PA to 7.2% of all-cause mortality among individuals aged ≥ 15 years^2^. Insufficient PA is also an independent predictor of cardiovascular diseases, which were associated with about 18.6 million deaths globally in 2019^3^. Sufficient PA during adolescence (i.e., engaging in an average of 60 min/day of moderate-to-vigorous, mostly aerobic PA across the week)^4^ promotes healthy body composition and improves cardio-metabolic profile and fitness^5,6^. Nevertheless, a pooled analysis of self-reported data from 1.6 million adolescents in 146 countries/territories documented the prevalence of physical inactivity (i.e., not engaging in sufficient PA) to be 81%^7^. Another study involving adolescents (*n* = 142,118) from 48 low- and middle-income countries (LMICs) reported the prevalence of sufficient PA to be 15.3%^8^.

The burden of physical inactivity among adolescents is unequally distributed across gender and socio-economic strata. Based on self-reported data, insufficient PA appears more prevalent among girls than boys in seven out of nine global regions (except South Asia and High-income Asia Pacific)^7^. Across Europe and North America, boys generally show higher prevalences of sufficient PA than girls, with gender differences ranging from 3.8 percentage points in Norway to 16.9 percentage points in Spain^9^. The odds of sufficient PA are 1.6 times higher among boys than girls in LMICs^8^. Furthermore, the socio-economic disparity in PA among adolescents differs in direction from that among adults. Insufficient PA among adult populations tends to increase with country income level^10,11^, whereas its prevalence among adolescents in high-income countries (HICs) is nearly 6% points lower than that in low-income countries^7^.

Similar to other health-related behaviors^12^, gender and socio-economic differences in PA become established in adolescence and contribute to inequalities in health outcomes later in life^13–15^. Hence, empirical evidence regarding gender and socio-economic differences in adolescents’ PA is of significant interest for tailoring public health initiatives. Nonetheless, existing literature on the relationship of socio-demographic attributes with PA during adolescence is riddled with critical limitations. The majority of the large-scale studies relied on PA self-reports^7,8,16,17^ that have well-known flaws: recall and social desirability biases^18^; reporting error associated with the type of PA^19^, length of recall period^20^, and respondents’ fitness level^21^; and limited insight on the validity and reliability of various adapted questionnaires. Accordingly, estimates of total and intensity-specific PA from questionnaire data are imprecise^22^. Moreover, studies employing PA questionnaires among adolescents often focus on dichotomous outcomes^7,8,16,17^ – sufficiently active or not, or meeting PA recommendation or not – and report prevalences and socio-demographic correlates of such outcomes. Consequently, estimates reflecting the absolute extent of gender and socio-economic differences in adolescents’ PA remain elusive. In addition, accelerometer-derived data from LMIC adolescents are scant as the vast majority of the studies utilizing accelerometers are from HICs^23^. Several accelerometer-based studies^24–26^ described time spent by adolescents in intensity-specific PA without controlling for socio-economic factors like parental education and socio-economic status. Thus, there are gaps in context-specific understanding of the gender and socio-economic differences in adolescents’ PA in resource-limited settings.

Physical fitness is another modifiable determinant of health that is interrelated with PA. Longitudinal studies have linked better fitness during adolescence to future health benefits such as lower adiposity, insulin resistance, and triglyceride levels^27^ as well as reduced all-cause premature mortality^28^. Both muscular and cardio-respiratory fitness have been shown to strongly track from mid-adolescence to well into adulthood^29,30^. However, an analysis of temporal trends among those aged 9–17 years from 19 high- and upper-middle-income countries revealed cardio-respiratory fitness declined by 7.3% between 1981 and 2014^31^. Gender disparity in adolescents’ fitness favouring boys is well established^32–35^, whereas previous research has produced contrasting findings when it comes to socio-economic differences. Studies among HIC adolescents generally demonstrate positive associations between socio-economic status and musculoskeletal and cardiorespiratory fitness^34–37^, but those from upper-middle-income countries capture inverse associations^33,38^. The contextual differences and methodological heterogeneity in assessing different fitness components (strength, endurance, flexibility, etcetera) limit generalizability of these findings to adolescents in resource-limited settings. Furthermore, there is a dearth of epidemiological studies from South Asia – home to the highest number of adolescents of any global region^39^ – that employed reliable, field-based fitness tests. The few published studies reported higher grip strength and aerobic capacity among boys than girls, yet did not examine socio-economic differences^40–42^.

Our previous study among Bangladeshi adolescents in a resource-limited setting^43^, demonstrated beneficial roles of habitual PA and muscular fitness in cardiometabolic health, stressing the importance of further understanding the socio-economic determinants of PA and fitness. In this context, we sought to explore the associations of gender, household wealth and maternal education with: (1) accelerometer-measured total and intensity-specific PA and sedentary time (ST), and (2) upper and lower body muscular and cardio-respiratory fitness at 15 years of age in a rural birth cohort from southeast Bangladesh. The specific research objective was to characterize gender-based and socio-economic inequalities in PA and fitness to inform context-specific public health strategies.

## Methods

### Participants and study setting

**Fig. 1:**
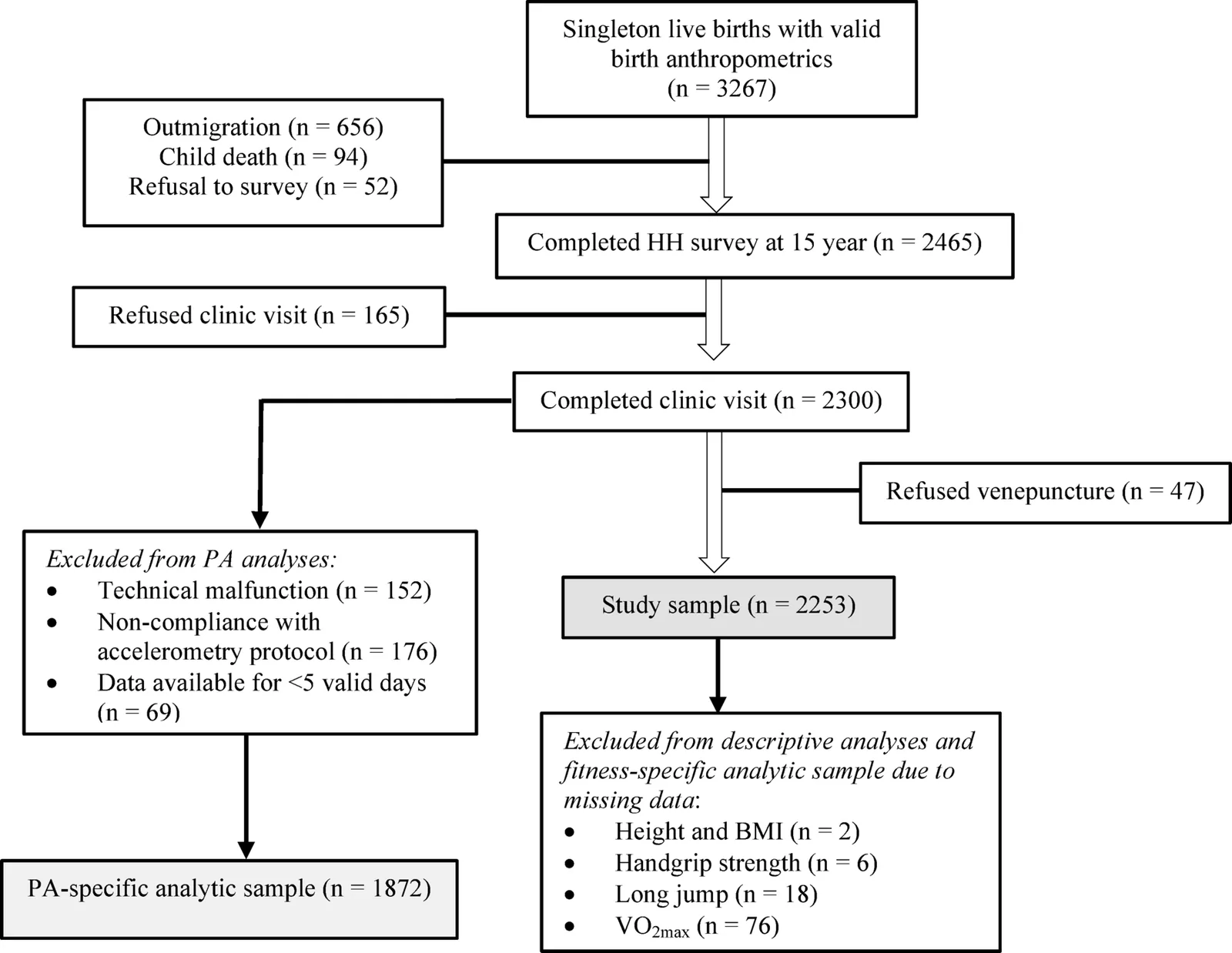
Flowchart for inclusion of MINIMat adolescents into the present study. Abbreviations: HH, household; PA, physical activity; BMI, body mass index; VO_2max_, maximal oxygen consumption.

This cross-sectional study was nested into the 15-year follow-up of MINIMat (Maternal and Infant Nutrition Interventions in Matlab) trial, conducted from September 2017 to June 2019. MINIMat (reg#ISRCTN16581394; registration date: 16 February 2009) was a community-based, randomized trial for testing the effects of prenatal food and micronutrient supplementation on maternal and birth outcomes^44^. A total of 4436 pregnant women were randomized between 2001 and 2003. There were 3625 live births, and 3267 of them were singletons with valid birth anthropometrics forming the MINIMat cohort that has been systematically followed up^44,45^. The 15-year follow-up comprised three modules: formative phase, household survey and clinic visit. Trained interviewers with ≥ 12 years of formal education interviewed the adolescent-mother/guardian dyads at their residences using a pre-tested, structured questionnaire. The clinic visit involved anthropometric and fitness assessments following a standardized protocol implemented by trained nurses, and the adolescents received accelerometers after their clinic visit. Figure 1 (Results section) outlines the participant flow into the present study. As details of the cohort design, follow-up procedures, and accelerometry and fitness test protocols have been published elsewhere^45,46^, only the key methodological elements are summarized here.

The study area of Matlab is a rural subdistrict in southeast Bangladesh with an agrarian community. While rice farming is the main occupation in Matlab, a few villages rely on fishing as the means of income^47^.

### Assessment of physical activity

Wrist-mounted, triaxial ActiGraph wGT3X-BT accelerometers (ActiGraph Corp, USA; dynamic range ±8G) were used to assess PA in free-living conditions. The participants wore a pre-tested accelerometer on their non-dominant wrist for at least five consecutive 24-hour periods (including night-time), except during water-based activities like swimming and showering/bathing. The devices were initialized to start collecting data from 12pm on the day they were fitted. The sampling frequency was 90 Hz. Data were processed and analyzed using the ActiLife software (version 6.13.4) in five-second epochs with the default ‘Normal filter’^48^ option. The five-second epoch suits the duration of PA bouts commonly observed among adolescents^49^, and provides sufficient resolution to avoid under-estimation of ST^50^ and vigorous PA^48^. Non-wear time (defined as ≥ 60 min of consecutive zero counts allowing non-zero interruptions of up to 2 min) and sleep time were deducted using algorithms validated among adolescents^51,52^. A valid accelerometry day entailed an awake wear time of ≥ 600 min, and participants accumulating at least five such valid days – including one weekend day – were retained in the analysis. We employed cut-points based on vector magnitude (VM, the square root of the sum of squared activity counts from the three axes) counts per epoch, validated by Chandler et al.^53^ to determine ST (≤ 305) and time engaged in intensity-specific PA: light-intensity (LPA: 306–817), moderate (MPA: 818–1968), vigorous (VPA: ≥1969), and moderate-to-vigorous (MVPA : ≥818). Total PA was assessed in terms of daily average of accumulated VM counts per minute. In the MINIMat cohort, we previously examined how accelerometer-measured total and intensity-specific PA relate to conventional markers of cardiometabolic risk^43,46^.

### Assessment of physical fitness

Muscular and cardio-respiratory fitness were assessed using: (1) handgrip strength, an indicator of upper body isometric strength; (2) standing long jump test, a proxy for lower body explosive strength; and (3) aerobic capacity or maximal oxygen consumption (VO_2max_) from a submaximal, multistage test – Chester Step Test^54–56^. We previously explored the cross-sectional associations of these parameters of physical fitness with cardiometabolic risk among adolescents of the MINIMat cohort^43,46^.

A digital, handgrip dynamometer (TKK 5401 Grip-D, Takei Scientific Instruments Company, Japan; range: 5–100 kg; accuracy: ±2 kg) was used to measure handgrip strength. The dynamometer’s grip-span was adjusted to the hand size of the participant. The adolescents stood with their arms completely extended and squeezed the dynamometer gradually to the maximum of their strength for at least 2 s^57^. The test was performed twice on each hand, alternating between the right and left side. The dynamometer was reset to zero after each attempt. The handgrip strength (kg) was derived as the mean of the highest readings from the right and left hands. To adjust for differences in body mass, weight-normalized grip strength was calculated by dividing handgrip strength with body weight (kg)^58,59^.

The standing long jump test^55^ was conducted indoors on a non-slip, hard surface. The adolescents stood behind a 50-cm long take-off line with their feet shoulder width apart, they pushed off vigorously and jumped as far forward as possible to land on both feet staying upright. The distance (cm) between the line and the heel mark of the foot closest to the line was measured. The maximum distance obtained from two attempts was used in the analysis.

Chester Step Test involved stepping on to and off a step, 30-cm high, at a rate set by a metronome – commencing with 15 steps/minute and increasing every two minutes by five steps/minute – with a heart rate monitor strapped to their chest^56^. The test continued until the adolescents reached 80% of the predicted maximum HR^60^[(220 − 15) × 0.8 = 164/minute], reported perceived exertion of 15 (“Hard”) on the Borg scale^61^, or completed all the five stages of the test. VO_2max_ (mL/kg/minute) was predicted using the CST software that incorporates a statistical line of best fit across the incremental HR recordings.

### Socio-demographic variables

Gender was a dichotomous variable (girl/boy). We adopted an asset-based approach to creating a proxy for socio-economic position that has been widely applied in LMICs^62^. Principal component analysis of data on ownership of selected durable articles (e.g., mobile phone, radio, television, refrigerator, bicycle, etcetera), access to electricity and sanitary latrine, and type of cooking fuel was performed. Using the factor score coefficients from the first principal component as weights, an asset score^60^ was constructed for each household. The asset scores were ranked in ascending order and divided into tertiles to categorize households into the poorest, middle, and richest groups according to relative household wealth. Educational status was assessed in terms of completed years of formal education, excluding schooling in an unregistered madrasa or evening school. Maternal education was categorized into: no education, primary (1–5 years), and secondary or above (≥ 6 years). Adolescents’ education was categorized into: no education, primary (1–5 years), and secondary (6–12 years). Due to the low number of adolescents with only primary education, this category was combined with that of no formal education during analysis.

### Anthropometric assessment

Body weight was recorded with a digital scale (Tanita BC-418 Body Composition Analyzer, 0.2 kg) and height with a stadiometer (Seca 214, 0.1 cm). Body mass index (BMI) was calculated by dividing body weight by height squared (kg/m^2^). BMI-for-age z-score (BAZ) was computed using the World Health Organization^63^. We categorized the adolescents as thin (BAZ < − 2), normal-weight (− 2 ≤ BAZ ≤ + 1), and overweight/obese (BAZ > + 1) for descriptive purposes.

### Statistical analysis

The analyses were performed in R (version 4.3.0). Sample descriptives are presented as frequency and percentage for categorical variables, and mean with standard deviation (SD) or median with interquartile range for numerical variables. We examined histograms and quantile-quantile plots for continuous variables to assess probability distribution and presence of outliers. Handgrip strength and VO_2max_ values > 5 SD of corresponding mean (*n* = 2 for handgrip; *n* = 6 for VO_2max_) were flagged implausible^64^ and excluded from the analyses. The Chester Step Test software excluded adolescents without adequate (i.e., at least two from the end of two completed stages) heart rate data points (*n* = 70) from the prediction of VO_2max_. Given the low proportion of missing data, we adopted a complete-case approach in the analyses. Weight-normalized grip strength was used in the regression analysis. Differences between groups at bivariate level were evaluated with Chi-squared test, independent samples t-test, or Wilcoxon rank-sum test. We fitted multiple linear regression models that simultaneously adjusted for gender, household wealth and maternal education. Models with ST and intensity-specific and total PA as outcomes were also adjusted for awake wear time. Predicted means from the models along with 95% confidence intervals (CIs) are reported. Distribution of the residuals and residuals versus fitted plots were checked to rule out violation of assumptions. A two-way interaction term incorporating gender and household wealth was tested and not found statistically significant in any model. Values of variance inflation factor were retrieved to rule out multi-collinearity and none exceeded 2.1.

## Results

Out of 3267 eligible adolescents, 2465 completed the household survey. Subsequently, 165 adolescents refused clinic visit and another 47 refused venepuncture. Thus, the study sample comprised 2253 adolescents. After excluding participants with invalid PA data due to technical malfunction, non-compliance and registering less than five valid accelerometry days; 1872 adolescents were retained in the PA analyses. The proportion of adolescents without any formal education was lower among those retained in PA analyses (17.1%) compared to those excluded (26.8%, *P* < 0.001). However, those retained and those excluded did not differ by gender, household wealth, maternal education, or BAZ. Adolescents with missing data on height, and hence, BMI (*n* = 2); handgrip strength (*n* = 6); long jump (*n* = 18); and VO_2max_ (*n* = 76) were excluded from analyses involving the respective variables (Fig. 1).

### Sample characteristics

The socio-demographic and anthropometric characteristics along with accelerometry and fitness profiles of the participants are presented in Table 1. The girls were shorter in stature and lighter in weight than the boys, but the median BMI was higher among the girls. Nearly 8% of the girls were overweight/obese. Thinness was more common among the boys than the girls.

The sample median awake wear time was approximately 937 min/day and it did not vary by gender. Five, six, and seven valid days of accelerometry data were available from 11%, 77%, and 10% of the participants, respectively. Total daily PA demonstrated a gender-based difference as the majority of the girls (36%) belonged to the bottom tertile of total PA, while the majority of the boys (38%) belonged to the top tertile. The adolescents spent about 69% of the daily awake wear time sedentary and about 7% in MVPA. These corresponded to a mean ST of 641 min/day and a median MVPA of 64 min/day, respectively. About 58% of the adolescents engaged in MVPA of 60 min/day or more, and this proportion did not differ by gender (57% of the boys and 59% of the girls; *p* = 0.317). The median levels of handgrip strength, standing long jump and VO_2max_ were higher among the boys than the girls as well (Table 1).

**Table 1:** Sample characteristics and descriptive physical activity and fitness profiles.

Characteristics	All (*n* = 2253)		Boys (*n* = 1079)		Girls (*n* = 1174)		*P*^1^ value
	*n*	Value	*n*	Value	*n*	Value	
Age (years)	2253	15.0 (0.1)	1079	15.0 (0.1)	1174	15.0 (0.1)	0.382
Height (cm)	2251	156.5 (7.6)	1078	160.5 (7.7)	1173	152.9 (5.4)	**< 0.001**
Weight (kg)	2253	43.9 (39.3–49.7)	1079	45.3 (39.3–51.0)	1174	43.1 (39.2–48.5)	**< 0.001**
BMI (kg/m^2^)	2251	17.8 (16.3–19.8)	1078	17.2 (15.9–18.9)	1173	18.4 (16.9–20.5)	**< 0.001**
BAZ categories^2^	2251						**< 0.001**
Normal	1649	73.3	707	65.6	942	80.3	
Thin	442	19.6	304	28.2	138	11.8	
Overweight/obese	160	7.1	67	6.2	93	7.9	
Household wealth	2253						**< 0.001**
Poorest	775	34.4	308	28.5	467	39.8	
Middle	723	32.1	397	36.8	326	27.8	
Richest	755	33.5	374	34.7	381	32.4	
Maternal education	2253						**0.027**
None	447	19.8	210	19.5	237	20.2	
Primary	800	35.5	357	33.1	443	37.7	
Secondary or above	1006	44.7	512	47.4	494	42.1	
Adolescent education	2253						**< 0.001**
None	355	15.8	214	19.8	141	12.0	
Primary	57	2.5	45	4.2	12	1.0	
Secondary	1841	81.7	820	76.0	1021	87.0	
Accelerometry characteristics							
Number of valid days	1872	6.0 (0.6)	887	6.0 (0.5)	985	6.0 (0.6)	0.254
Awake wear time (min/day)	1872	936.6 (874.6–991.7)	887	933.8 (871.1–991.0)	985	938.8 (877.7–992.0)	0.399
Total daily PA (VMCPM/day)	1872	320.4 (275.9–371.2)	887	329.5 (279.7–379.8)	985	312.9 (273.3–361.8)	**< 0.001**
Tertile 1 (< 289.9)	624	33.3	269	30.3	355	36.1	
Tertile 2 (289.9–350.1)	625	33.4	279	31.5	346	35.1	
Tertile 3 (> 350.1)	623	33.3	339	38.2	284	28.8	
ST (min/day)	1872	641.2 (92.0)	887	635.3 (93.0)	985	646.5 (90.9)	**0.008**
ST percentage^3^	1872	69.1 (65.1–73.2)	887	68.6 (64.4–72.9)	985	69.4 (65.9–73.4)	**0.002**
LPA (min/day)	1872	220.5 (42.4)	887	224.3 (45.1)	985	217.2 (39.4)	**< 0.001**
Moderate PA (min/day)	1872	64.6 (22.6)	887	62.9 (23.0)	985	66.2 (22.2)	**0.001**
Vigorous PA (min/day)	1872	1.0 (0.4–2.6)	887	2.4 (1.1–4.8)	985	0.5 (0.2–0.9)	**< 0.001**
Vigorous PA percentage	1872	0.1 (0.04–0.3)	887	0.3 (0.1–0.5)	985	0.05 (0.03–0.1)	**< 0.001**
MVPA (min/day)	1872	64.3 (49.9–80.9)	887	63.8 (48.1–81.5)	985	64.5 (51.2–80.4)	0.270
MVPA percentage^3^	1872	6.9 (5.4–8.7)	887	6.8 (5.3–8.7)	985	7.0 (5.6–8.6)	0.363
Fitness characteristics							
Handgrip strength (kg)	2247	24.4 (21.1–29.8)	1078	30.0 (25.3–33.7)	1169	21.7 (20.0–24.0)	**< 0.001**
Weight-normalized grip strength^4^	2247	0.58 (0.12)	1078	0.65 (0.10)	1169	0.51 (0.09)	**< 0.001**
Standing long jump distance (cm)	2235	141.0 (124.0–162.0)	1071	162.0 (148.0–175.0)	1164	125.8 (115.8–136.2)	**< 0.001**
VO_2max_ (mL/kg/min)	2177	34.5 (31.0–37.3)	1057	35.1 (31.8–38.2)	1120	33.8 (30.3–36.6)	**< 0.001**

BMI, body mass index; BAZ, BMI-for-age z-score; PA, physical activity; VMCPM, vector magnitude counts per minute; ST, sedentary time; LPA, light-intensity physical activity; MVPA, moderate-to-vigorous physical activity; VO_2max_, maximal oxygen consumption. Values represent percentage, mean (standard deviation), or median (inter-quartile range), as appropriate. Missing data: height and BMI (n = 2), handgrip strength and weight-normalized grip strength (n = 6), standing long jump (n = 18), VO_2max_ (n = 76). ^1^P value for gender difference from Chi-squared test, independent samples t-test, or Wilcoxon rank-sum test. ^2^BAZ below –2 SD and above +1 SD define thinness and overweight/obesity, respectively. ^3^Percentage of daily awake wear time spent sedentary and in moderate-to-vigorous physical activity. ^4^Derived by dividing participant’s handgrip strength (kg) by his/her body weight (kg). P values in bold indicate statistically significant differences.

### Gender and socio-economic differences in ST and total and intensity-specific PA

**Table 2:** Estimated durations of sedentary time and total and intensity-specific physical activity from multiple linear regression models.

Variable	ST (min/d)	Total PA^1^	LPA (min/d)	MPA (min/d)	VPA (min/d)	MVPA (min/d)
	Predicted^2^ mean (95% CI)					
Gender						
Girl	644.2 (640.6–647.7)	322.7 (317.6–327.7)	217.0 (214.6–219.4)	66.4 (65.0–67.8)	0.8 (0.6–0.9)	67.2 (65.7–68.7)
Boy	635.2 (631.5–638.9)	338.3 (333.0–343.6)	226.0 (223.5–228.6)	63.7 (62.2–65.2)	3.5 (3.3–3.6)	67.1 (65.6–68.7)
P value	**< 0.001**	**< 0.001**	**< 0.001**	**0.007**	**< 0.001**	0.973
HH wealth						
Poorest	631.0 (626.7–635.3)	341.0 (334.9–347.2)	227.8 (224.9–230.7)	67.4 (65.7–69.1)	2.2 (2.0–2.4)	70.0 (67.8–71.4)
Middle	640.3 (635.9–644.7)	326.8 (320.5–333.1)	220.9 (217.9–223.9)	65.1 (63.3–66.8)	2.1 (2.0–2.3)	67.2 (65.3–69.0)
Richest	647.8 (643.0–652.6)	323.6 (316.7–330.5)	215.9 (212.6–219.2)	62.7 (60.8–64.6)	2.0 (1.8–2.2)	64.7 (62.7–66.7)
P^**3**^ value	**0.003**	**0.002**	**0.001**	0.067	0.503	0.071
P^**4**^ value	**< 0.001**	**< 0.001**	**< 0.001**	**< 0.001**	0.145	**< 0.001**
Maternal education						
None	635.8 (630.1–641.5)	338.3 (330.1–346.5)	223.8 (219.9–227.7)	66.5 (64.2–68.8)	2.3 (2.1–2.5)	68.8 (66.4–71.2)
Primary	637.1 (632.9–641.4)	332.8 (326.7–338.9)	223.1 (220.3–226.0)	65.9 (64.2–67.5)	2.2 (2.1–2.4)	68.1 (66.3–69.9)
Secondary/higher	646.1 (642.2–650.0)	320.3 (314.8–325.8)	217.7 (215.1–220.3)	62.8 (61.2–64.3)	1.8 (1.7–2.0)	64.6 (63.0–66.2)
P^**5**^ value	0.704	0.283	0.787	0.648	0.719	0.639
P^**6**^ value	**0.005**	**< 0.001**	**0.014**	**0.010**	**0.003**	**0.006**

ST, sedentary time; PA, physical activity; LPA, light-intensity physical activity; MPA, moderate-intensity physical activity; VPA, vigorous-intensity physical activity; MVPA, moderate-to-vigorous physical activity; min/d, minutes per day; CI, confidence interval. ^1^In terms of vector magnitude counts per minute accumulated per day. ^2^Predicted from multiple linear regression models adjusted for gender, household wealth, maternal education, and awake wear time (minutes/day). Maternal education was assessed with completed years of formal education: none, primary (1–5 years) and secondary or higher (≥6 years). ^3^For the difference between poorest and middle categories.^4^For the difference between poorest and richest categories. ^5^For the difference between no formal education and primary education. ^6^For the difference between no formal education and secondary/higher education. P values in bold indicate statistically significant differences. Adjusted R² values for the underlying regression models were: 0.65 (sedentary time), 0.03 (total activity), 0.24 (light-intensity activity), 0.08 (moderate activity), 0.27 (vigorous activity), and 0.07 (moderate-to-vigorous activity).

Table 2 demonstrates the predicted means with 95% CIs for daily ST, total PA, and time engaged in intensity-specific PA by categories of gender, household wealth and maternal education. Gender displayed independent associations with ST, total PA, and intensity-specific PA apart from MVPA. The girls, on average, spent 9 min/day more being sedentary than the boys (*p* < 0.001); and engaged in 9 min/day less in LPA (*p* < 0.001) and 3 min/day less in VPA (*p* < 0.001) than the boys. However, the level of daily MPA was nearly 3 min higher among the girls (*p* = 0.007).

Daily ST was approximately 17 min higher among adolescents from the richest households compared to their peers from the poorest households (*p* = 0.003). Levels of total PA and LPA were higher among adolescents from the poorest households than among adolescents from the middle-status or richest households. Those from the poorest households engaged in approximately 5 min more MVPA daily than those from the richest households (*p* < 0.001). There was no association between household wealth and VPA. The difference in ST between those born to mothers with the highest educational attainment and those having mothers without formal education surpassed 10 min/day (*p* = 0.005). The latter also had higher total PA, and engaged more in PA of all intensities than those born to mothers with secondary or higher education (Table 2).

### Gender and socio-economic differences in muscular and cardio-respiratory fitness

**Table 3:** Estimated levels of grip strength, distance obtained from standing long jump and maximal oxygen consumption from multiple linear regression models.

Variable	Weight-normalized grip strength^1^	Standing long jump^2^ (cm)	Maximal oxygen consumption^2^ (mL/kg/min)
	Predicted^3^ mean (95% CI)		
Gender			
Girl	0.51 (0.50–0.51)	126.3 (125.4–127.2)	33.2 (32.9–33.6)
Boy	0.66 (0.65–0.66)	160.2 (158.9–161.4)	34.8 (34.5–35.2)
P value	**< 0.001**	**< 0.001**	**< 0.001**
Household wealth			
Poorest	0.59 (0.59–0.60)	143.5 (142.3–144.8)	34.3 (34.0–34.7)
Middle	0.59 (0.58–0.60)	142.2 (140.9–143.5)	34.0 (33.6–34.3)
Richest	0.56 (0.55–0.57)	140.9 (139.5–142.3)	33.8 (33.4–34.2)
P^**4**^ value	0.447	0.156	0.168
P^**5**^ value	**< 0.001**	**0.008**	0.062
Maternal education			
None	0.58 (0.57–0.59)	141.8 (140.1–143.5)	34.1 (33.6–34.7)
Primary	0.59 (0.58–0.59)	143.2 (141.9–144.4)	34.1 (33.7–34.5)
Secondary/higher	0.58 (0.57–0.58)	141.7 (140.6–142.8)	33.8 (33.5–34.2)
P^**6**^ value	0.056	0.210	0.931
P^**7**^ value	0.926	0.891	0.348

CI, confidence interval. Maternal education was assessed in terms of completed years of formal education: none, primary (1–5 years) and secondary or higher (≥6 years). ^1^Derived by dividing participant’s handgrip strength (kg) by his/her body weight (kg).^2^For these two right-skewed variables, the predicted means represent geometric mean. ^3^Predicted from multivariable linear regression models adjusted for gender, house-hold wealth and maternal education. ^4^For the difference between poorest and middle-status categories. ^5^For the difference between poorest and richest categories. ^6^For the difference between no formal education and primary education. ^7^For the difference be-tween no formal education and secondary or higher education. P values in bold indicate statistically significant differences. Adjusted R² values for the underlying regression models were 0.40 (weight-normalized grip strength), 0.48 (standing long jump), and 0.02 (maximal oxygen consumption).The three indicators of fitness were previously used to examine cross-sectional associations of fitness with selected markers of cardiometabolic risk in this study population^43^

The predicted means of weight-normalized grip strength, standing long jump and maximal oxygen consumption by gender, household wealth and mothers’ educational attainment from multiple linear regression models are presented in Table 3. After accounting for household wealth and maternal education, boys demonstrated higher levels of muscular and cardio-respiratory fitness than girls. Based on the predicted means, grip strength relative to body weight was approximately 29% higher among boys compared with girls (*p* < 0.001). On average, jump distances registered by the boys were 34 cm higher (*p* < 0.001) and VO_2max_ levels in boys were 1.6 mL/kg/min higher (*p* < 0.001) than in girls. Across household wealth tertiles, there were small but statistically significant differences between adolescents from the poorest and wealthiest households in grip strength (*p* < 0.001) and jump distance (*p* = 0.008). We did not observe any statistically significant association between maternal education and muscular or cardio-respiratory fitness.

## Discussion

We observed gender and socio-economic differences in PA and fitness that put the girls and adolescents from the wealthy households at a considerable disadvantage. The girls recorded higher ST, engaged less in LPA and VPA, and had appreciably lower muscular fitness than the boys. However, the gradient was reversed for MPA as its level was slightly higher among the girls, and no gender-based difference in MVPA was observed. Adolescents from the well-off households were substantially more sedentary and less active with regard to total PA, LPA and MPA. Corresponding to this socio-economic gradient in PA, we found adolescents form the poorest households to have better muscular fitness than their peers from the richest households. Maternal educational imparted an unhealthy influence on adolescents’ movement behavior, displaying a positive association with ST and a negative association with MVPA. The socio-economic gradient in PA appeared more pronounced than the gender difference in PA, whereas the magnitude of gender difference in muscular fitness was larger than that of the socio-economic difference.

The gender-based difference in PA observed in our study agrees with several accelerometer-based studies^24–26,65,65,67^, although only two of these studies^66,67^ adjusted for socio-demographic variables similar to ours. After controlling for wealth, maternal education and wear time, daily ST exceeded 10 h in this study: 644 min among the girls and 635 min among the boys. These ST estimates are noticeably higher than those reported by Dalene et al.^67^(587 min among girls, 566 min among boys at 15 years), but similar to those documented by Nyberg et al.^66^(644 min among girls, 613 min among boys at 14–15 years). Dalene et al.^67^ opted for 10-sec epochs; but shorter, five-second epochs were used by Nyberg et al.^66^ and in our study giving better ‘resolution’ for capturing ST^68^. Nyberg et al.^66^ found 3 min/day higher MPA among boys at 14–15 years, whereas MPA was 3 min/day higher among the girls in our study. However, in line with the 5 min/day higher VPA level among boys observed by Nyberg et al.^66^, VPA was 3 min/day higher among the boys in our study. The higher MPA level among girls might be attributed to a gendered labour division in a rural, agrarian setting that assigns domestic chores like sweeping, washing, homestead gardening, etcetera to adolescents girls^11,69^. These household activities are likely to have been classified as MPA^70^ in our study. Conversely, boys in Matlab bike^69^, play sports like football and cricket – popular in resource-constrained communities^11,69^– and often help their fathers in agricultural activities during cultivation and harvesting seasons^69^. Thus, the higher VPA observed among MINIMat boys was an expected finding. Socio-cultural aspects have been frequently identified in qualitative literature as drivers of gender-based differences in PA. Interestingly, focus groups involving adolescents in Matlab^69^ and in disadvantaged areas of Northwest England and Ireland^71^ converge regarding: (1) social stereotyping of what type of PA is ‘expected’ among adolescent girls; (2) lack of social acceptance for girls engaging in health-promoting activities like biking and playing football; and (3) perception among girls that they lack the skills and stamina required for strenuous sports^69,71^. Nonetheless, in contrast to previous studies^65,65,67^, we did not observe a gender-based difference in MVPA that essentially aggregates MPA and VPA. Of note, those studies that found a difference used much lower cut-points for capturing MVPA than the Chandler^53^ cut-point that we used: ≥2296^65,66^ and ≥ 2000^67^ counts/minute.

In parallel to the gender-based difference in PA, boys were fitter than the girls in our study. Higher fitness levels among adolescent boys has been documented in other large, observational studies^32,34,36^ too. The magnitude of difference in muscular fitness in this study was larger than that reported by Sandercock et al.^34^ among Columbian adolescents aged 14–16 years after controlling for similar socio-economic variables. The gender-based difference in muscular fitness among the MINIMat adolescents could have stemmed from lower VPA levels and lack of sports participation among the girls. In fact, current literature persistently links sports participation with higher muscular fitness among adolescents^72^. It is also plausible that shorter stature^34^ and lower lean mass^73^ among girls contributed to the substantial gender-based difference in the standing long jump test. Median fat-free mass, estimated from bioelectrical impedance, was 26.8 kg among girls and 33.2 kg among boys of the MINIMat cohort at 15-year follow-up (data not shown). Independent of wealth and maternal education, we observed 1.6 mL/kg/minute higher VO_2max_ among the boys. Although this is a modest difference as 1 MET (metabolic equivalent of tasks) represents 3.5 mL/kg/minute oxygen consumption^74^, difference in adolescents’ VO_2max_ by gender tends to peak at 17 years of age and onwards^75^. Descriptive studies from elsewhere also demonstrated higher VO_2max_ among boys^32,35^, albeit without adjusting for socio-economic variables.

In this study, socio-economically disadvantaged adolescents – from the poorest households and those having mothers without any formal education – were less sedentary and more active than their peers from wealthier households. This socio-economic gradient in Matlab contradicts the pro-rich disparity in adolescents’ PA reported from HICs^16,76,77^. In high-income settings, the higher PA level among adolescents of affluent families is largely driven by their participation in recreational, leisure-time PA including organized sports^78,79^. Participation in structured, leisure-time activities requires the families to meet additional costs like gymnasium or club membership fees, equipment and transport cost, etcetera^79^. A recent meta-analysis pooling studies from HICs found adolescents from higher socio-economic backgrounds were 1.8 times more likely to participate in organised sports than their worse-off peers^80^. Adolescents from affluent families in HICs are more likely to commute actively to/from school (biking or walking)^81^ and one-way active commute can contribute 5–26 min of MVPA (up to 48% of the daily recommendation)^82^. Nevertheless, these explanations for the pro-rich disparity in high-income settings are not applicable to the rural context of Matlab where opportunities for organised sports or gymnasium-based, focused PA are lacking^69^. Instead, higher PA among disadvantaged adolescents in Matlab might be reflective of their greater engagement in economic activities to support their families. These activities usually involve works related to farming, poultry rearing and tending to livestock^69,83^. This interpretation remains speculative as information on the context and domains of PA was unavailable. Further research is needed to understand how movement behaviors contributing to intensity-specific PA and sedentary bouts vary by socio-economic factors in resource-limited, rural settings. We also observed better levels of muscular fitness among adolescents belonging to the poorest households. This aligns with findings from upper-middle-income countries like Colombia^33^ and Brazil^38^, where wealth or income demonstrated an inverse association with muscular fitness among adolescents. The heterogeneity in empirical evidence regarding the link between family affluence and physical fitness of adolescents necessitates well-designed studies to identify contextual factors underlying these variations.

While maternal education may promote awareness of health benefits from PA^79^, MINIMat adolescents of educated mothers had higher ST and lower total and intensity-specific PA. A pooled analysis of eight accelerometer studies from HICs also revealed 9.5 min/day higher ST and 10 min/day lower LPA among adolescents of university-educated mothers versus those with high-school-educated mothers^84^. Among Swedish adolescents aged 14–15 years, higher parental education (> 12 years, either parent) was associated with 19 min/day higher ST compared to lower parental education (≤ 12 years) regardless of age, sex, municipality and birth country^66^. We posit that educated mothers in Matlab might have prioritized academic achievements over sports or recreational PA for their children^11^ which potentially led to more ST and less active movement behaviors^85^. Nonetheless, maternal education was not associated with muscular or cardiorespiratory fitness, presumably reflecting a time lag before differences in fitness parameters by maternal education become apparent.

Implications of the gender-based and socio-economic differences that we observed should be evaluated against effect sizes from studies on the relationship of PA and fitness with cardio-metabolic parameters. ST differed by 9–17 min/day, LPA by 9–12 min/day, and MVPA by 4–5 min/day across gender, wealth and maternal education categories in our analyses. In the same sample, we reported^43^ 10 min/day higher ST was associated with 0.1% higher waist circumference and 0.9% higher insulin resistance. Moreover, 10 min/day higher MVPA was associated with 0.4% lower waist circumference and 1.9% lower insulin resistance. In contrast, LPA in itself was not associated with any cardiometabolic marker, but equivolume substitution of ST with LPA was associated with 1.4% lower insulin resistance^43^. In a birth cohort from England, 1 min/day higher ST led to 1.3 g higher total fat mass and 1 min/day higher MVPA lowered it by the same amount over the age period from 11 to 24 years^86^. Some studies among adolescents also indicate LPA can slightly attenuate the association of ST with body adiposity^87^ and 60 min/day of LPA is associated with 0.6 mmHg lower diastolic blood pressure^88^. Among adolescents, the magnitude of favorable association with cardiometabolic markers is notably higher for VPA than LPA. Similarly, meta-analyses of correlation coefficients (r) indicated small to moderate effect sizes for prospective associations of adolescents’ muscular fitness with insulin resistance (*r* = − 0.1), triglyceride (*r* = − 0.22), and composite cardio-metabolic risk score (*r* = − 0.29)^27^. Inferring from these estimates, cardio-metabolic implications of the gender and socio-economic patterning may appear inconsequential around mid-adolescence. Nevertheless, the differences in PA are likely to increase with age reaching critical levels of public health relevance in ensuing adulthood^89^. Similar to the tracking of PA patterns^90^, handgrip and lower body strength both track strongly from mid-adolescence up to two decades later^29^. If gender and socio-economic differences apparent at mid-adolescence follow this trajectory, these would drive significant disparities in long-term cardio-metabolic outcomes. Thus, PA and fitness interventions for adolescents that take into account existing gender and socio-economic differences are imperative to address the rising burden of cardio-metabolic disorders^91^ in resource-constrained LMIC settings. However, little is known about effective implementation of community-based PA interventions for adolescents as a recent systematic review identified only six intervention studies among adolescents in rural and remote communities with negligible impacts^92^.

Key strengths of this study were: objective assessment of PA and fitness among adolescents of a rural birth cohort leveraging a long-standing community research infrastructure^47^; field implementation of reliable, cost-effective fitness tests in a low-income setting; high-quality data on socio-economic factors that rarely serve as primary variables of interest in accelerometer studies, and stringent analytical criteria for PA (≥ 10 h/day of accelerometry combined with ≥ 5 valid days including one weekend day) to closely represent habitual PA. The PA intensity cut-points employed in this study were developed using ActiGraph devices worn on the non-dominant wrist and employing five-second epochs^53^. We used the same accelerometer placement and epoch length to obtain reliable estimates, as recommended in the literature^48^. Given the area-wide recruitment of pregnant women in the MINIMat trial, the findings are generalizable to adolescents in Matlab. This can be extended to rural adolescents in other agrarian settings of Bangladesh because of the socio-demographic and cultural similarities. Our results, however, need to be interpreted in light of certain limitations. The cross-sectional design precluded temporality and drawing causal inferences from the associations would be erroneous. Residual confounding could not be entirely ruled out as well. A potential source of misclassification relates to the algorithmic exclusion of non-wear time as it is impossible to distinguish ST or sleep from non-wear time with 100% certainty^93^. Furthermore, a noteworthy limitation of accelerometer-based assessment of PA is that estimates from studies adopting different epoch lengths and intensity cut-points are not directly comparable^48^. Although Chester Step Test offers simplicity and cost-effectiveness for epidemiological studies in resource-limited settings, its precision in estimating VO_2max_ appears lower than that of cycle ergometers or treadmills^94^.

## Conclusion

This study indicated the presence of considerable gender and socio-economic differences in objectively measured PA and fitness at 15 years of age in a birth cohort of rural adolescents from Bangladesh. Girls and adolescents from affluent families were more sedentary, less active, and had worse muscular fitness compared to boys and adolescents from poor households, respectively. Higher maternal education was linked to a less favorable PA profile with higher ST and lower total and intensity-specific PA. Addressing these gender and socio-economic disparities will require locally informed public health initiatives. To identify potential avenues for interventions, future work should examine behavioral and contextual factors – such as duration of ST bouts, mode of travel to and from school, and the rural built environment – that may underlie these differences.

## Data Availability

We utilized data from the 15-year follow-up of the MINIMat (Maternal and Infant Nutrition Interventions in Matlab) trial—a large, collaborative project involving Uppsala University, Karolinska Institute, Finnish Institute for Health and Welfare, University of Oulu, and icddr, b. Because of the statutory requirements, internal data policies and regulations existing in the collaborating bodies along with the over-arching General Data Protection Regulation (GDPR), the data must be stored in institutional repository (storage platforms) and cannot be made directly accessible without a review of the request for access. Data availability is further limited because the data contain information on gender and health-related and behavioral attributes, and thus, considered to be “sensitive personal data” as per GDPR. While the data are pseudonymized in accordance with GDPR, attributes that can link the data to each study participant exist and are preserved following regulations in place at the collaborating bodies. Therefore, the data can be accessed only upon a formal request that details the purpose of such request. Any request to access should be directed to: E-CE (email: lotta.ekstrom@uu.se), and AR (arahman@icddrb.org).
